# Supratentorial primitive neuroectodermal tumor in an adult: a case report and review of the literature

**DOI:** 10.1186/1752-1947-6-361

**Published:** 2012-10-24

**Authors:** Shokry Lawandy, Omid R Hariri, Dan E Miulli, Jenny Amin, Tanya Minasian, Ravi K Gupta, Javed Siddiqi

**Affiliations:** 1 Department of Neurosurgery, Arrowhead Regional Medical Center, 400 North Pepper Ave, Colton, CA 92324, USA; 2 Department of Surgery, College of Osteopathic Medicine, Western University of Health Sciences, 309 E. 2nd St, Pomona, CA 91766, USA; 3 Department of Radiology, Harbor-UCLA Medical Center, 1000 West Carson Street, PO Box 2910, Torrance, CA 90509, USA

## Abstract

**Introduction:**

Supratentorial primitive neuroectodermal tumors predominantly occur in children, and are rare in the adult population. Less than 100 cases of supratentorial primitive neuroectodermal tumor have been reported in adults internationally. Our case study reports this rare incident.

**Case presentation:**

A 22-year-old Hispanic man presented with headaches, blurry vision, diplopia, intermittent vomiting, and grossly decreased vision. A magnetic resonance image showed a left posterior parietal heterogeneously enhancing mass measuring 4.2cm × 7.2cm × 7.0cm. After craniotomy for resection and decompression, the mass was histologically revealed to be a supratentorial primitive neuroectodermal tumor. Standardized immunohistochemical studies for this mass were carried out.

**Conclusion:**

We have concluded that immunohistochemical and genetic workup should be included in the standardized pathological workup for primitive neuroectodermal tumors in order to provide more prognostic information. Based on our current literature review, we propose an immunohistochemical panel.

## Introduction

Supratentorial primitive neuroectodermal tumors (sPNETs) are known to be tumors of the pediatric population. According to the World Health Organization, sPNETs are described as a cerebral or suprasellar embryonal grade IV tumor made up of undifferentiated or poorly differentiated neuro-epithelial cells which have the capacity for, or display, divergent differentiation along neuronal, astrocytic, ependymal, muscular or melanocytic lines. Approximately 1% of pediatric brain tumors are sPNETs. However, they can sporadically occur in adults. To date, only less than 100 sPNET cases have been reported in adult patients [1].

In an extensive case review series by Ohba *et al.*, it was determined that the mean age of diagnosis in adults was 35.2 years of age, with one peak between the ages of the second and third decades. Throughout all age groups, more males were prone to be diagnosed with pNETS than females [1].

Of the PNETs in the central nervous system (CNS) 5.6% are supratentorial. Moreover, the locations of these tumors are almost equally distributed in the frontal, temporal, and parietal lobes [1]. Less than 50% of these patients have a survival rate of 5 years post-diagnosis [2]. Radiographically, the best diagnostic clue is the presence of a large, complex hemispheric mass with minimal peritumor vasogenic edema. Cerebral hemispheric PNETs have a mean diameter of approximately 5cm at diagnosis. PNETs often appear with necrosis, intra-tumor hemorrhage, cysts, and calcification (50–70%). On computed tomography, these lesions appear as isodense or hyperdense. On magnetic resonance imaging (MRI), these tumors appear as well-delimited, inhomogeneous, and variably contrast-enhanced lesions. Furthermore, on T1-weighted images, PNETs appear hypointense, and on T2-weighted images they appear hyperintense [3-6].

**Figure 1:**
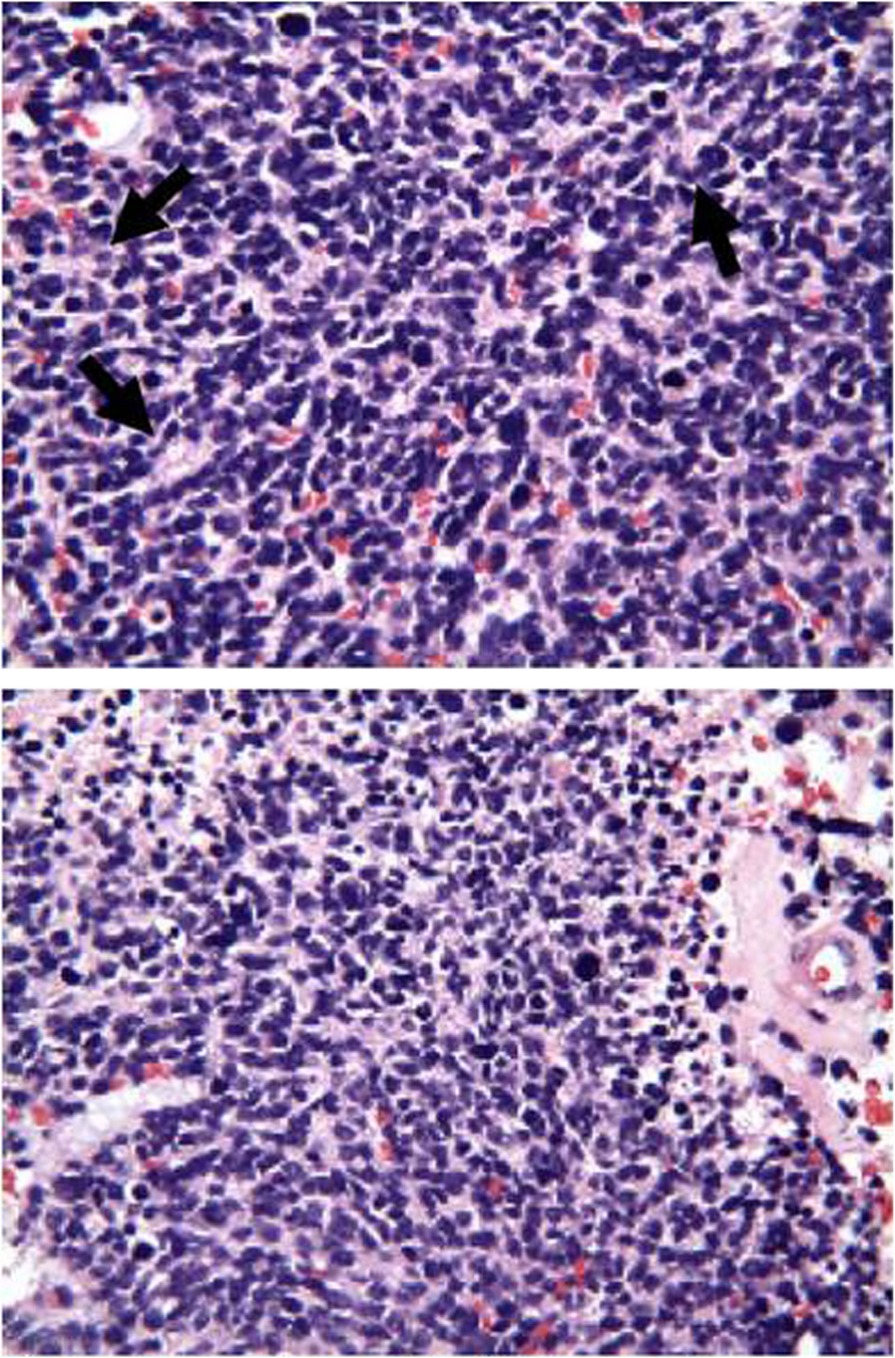
**Histopathology. **Photomicrographs of tumor showing small, round, and undifferentiated cells with scant cytoplasm and round or oval nuclei with dense chromatin or prominent nucleoli, forming structures reminiscent of Homer-Wright rosettes (black arrowheads), as well as mitosis.

Histologically, the highly cellular tumor consists of anaplastic cells with small round to oval hyperchromatic nuclei surrounded by scanty cytoplasm (Figure 1). After a review of the literature, we propose a novel pathological and genetic panel workup for brain masses in adults that are suspected to be PNETs based on radiographic studies and intra-operative pathological diagnosis.

## Case presentation

A 22-year-old Hispanic man was referred to a neurosurgery clinic by his ophthalmologist, who was concerned about increased intracranial pressure after noticing papilledema on fundoscopic examination. The patient had been complaining of headaches, blurry vision, diplopia, and intermittent vomiting for a month. He reported that his symptoms had been worsening over the past week. The patient has no other significant past medical history and has been in general good health besides the recent onset of headaches. He was a resident of California. Furthermore, on physical examination, left horizontal nystagmus and grossly decreased vision on the right temporal and right nasal hemifield were noticed.

**Figure 2:**
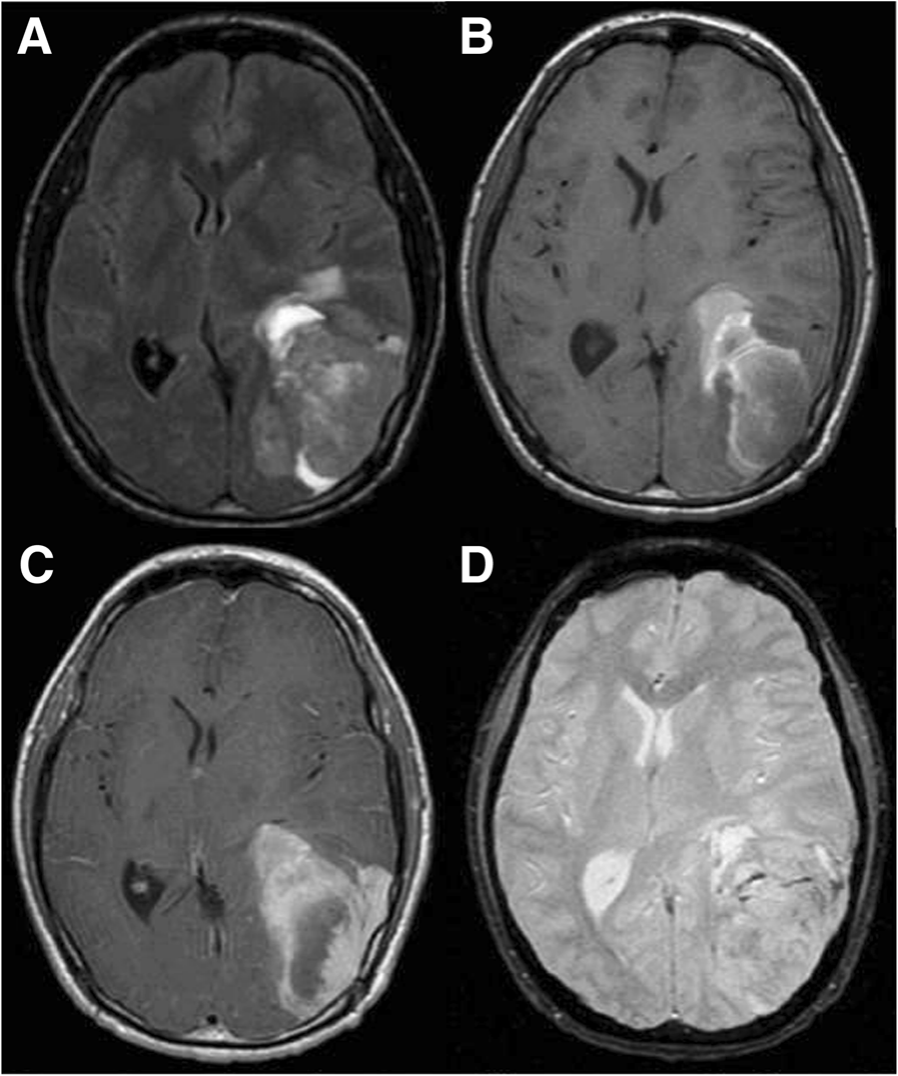
**Magnetic resonance image of mass. A**: Flair showing mild peritumoral edema, **B**: T1-weighted image without contrast, **C**: T1-weighted image with contrast showing homogenous enhancement, **D**: Gradient echo.

A brain MRI revealed a left posterior parietal, irregular, hemorrhagic mass measuring approximately 4.2cm × 7.2cm × 7.0cm, enhancing heterogeneously, and associated surrounding vasogenic edema and a 8.7mm midline shift from left to right. Compressions of the atrium of the left lateral ventricle, and the third ventricle, as well as some effacement of the quadrigeminal plate cistern, particularly on the left were noted (Figure 2).

The patient underwent a left parieto-occipital craniotomy with resection, and the ultimate goal of debulking of mass. Intra-operatively, the lesion was noted to have a necrotic center. Initially, a fresh frozen specimen showed the tumor to be glioblastoma multiforme. However, a studied permanent specimen indicated a primitive neuroectodermal tumor with 5 to 10 mitoses per high-power field. There were many cells immunoreactive to neuron-specific enolase, CD56, and small cell neuroendocrine carcinoma. However, none were immune-reactive to synaptophysin or glial fibrillary acidic protein. There were no intra-operative or postoperative complications.

## Discussion

Therapeutic approaches are close to non-existent because the pathological mechanisms underlying sPNETs are poorly understood. Some of the proposed genetic alterations include: isochromosome 17q; losses of chromosomes 6q, 9q, 10q, 11, and 16q; trisomy-1q; and mutations of *TP53*. PNETs are classified into peripheral and central. Intracranial peripheral PNET has a better prognosis in comparison to central PNET. Accordingly, it is essential to differentiate between the two types. Chromosomal translocation of chromosome (11;22) is phenomenal in that it is unique to central and not peripheral PNETs. Thus, the usage of immunohistochemical assay for CD99 and fluorescence *in situ* hybridization (FISH) assay for the (11;22) translocation have granted the capability to distinguish between intracranial peripheral and intracranial central PNET [1,7]. More importantly, in a study by Hayden *et al.*, it has been suggested that mutations in *IDH1*, which encodes for cytoplasmic isocitrate dehydrogenase, are among the most frequent mutations in sPNET, and are much more prevalent in adult cases in comparison to pediatric cases [8]. Furthermore, Gessi *et al*. have shown that a high incidence of *TP53* mutations and the absence of amplification of the *c-myc/N-myc* genes in adult sPNET, in comparison to pediatric PNET, suggest that adult sPNET should be an independent subset of tumors among CNS-PNETs [3].

Even though no prospective treatment studies have been conducted to date, and no optimal treatment regimen for sPNET has been established, a complete surgical excision followed by radiation therapy has been proved to be crucial in many retrospective studies [9-12]. Radiotherapy to the entire neuroaxis is recommended because cerebrospinal dissemination at the time of diagnosis was found in almost 10% of all cases.

## Conclusion

An extensive review of the current literature suggests that immunohistochemical and genetic assays are becoming essential markers for prognosis and possible treatment of sPNETs. As we discover more about all the mentioned studies, it will become essential to integrate them into our standard pathological workup for brain masses in adults that are highly suspicious to be PNETs based on radiographic studies and preliminary intra-operative pathological diagnosis.

We propose immunohistochemical and genetic assays panels, which to the best of our knowledge has not been proposed in the literature before, and we have decided to use this panel in our institutions. We believe that initially a genetic assay should evaluate any potential mutations to *IDH1* (more prevalent in adult cases) to differentiate adult sPNET from other possible pediatric PNETs. Furthermore, an evaluation of *TP53* mutations (high incidents in adults) and *c-myc/N-myc* genes (absence of amplification in adults) should be evaluated in order to differentiate between a true sPNET and any pediatric PNETs. Subsequently, a CD99 and a FISH assay for the (11;22) translocation (phenomenal in that it is unique to central and not peripheral PNETs) should be conducted in order to distinguish between intracranial peripheral and intracranial central PNETs, because the prognosis of central and peripheral PNETs are drastically different, as mentioned previously.

## Consent

Written informed consent was obtained from the patient for publication of this case report and accompanying images. A copy of the written consent is available for review by the Editor-in-Chief of this journal.

## Competing interests

The authors declare that they have no competing interests.

## Authors’ contributions

SL was the physician for the patient in the manuscript and was a contributor in writing the manuscript. OH was a major contributor in writing the molecular neurobiology segments of the manuscript, as well as the main editor. DM was the chief editor of this manuscript. JA and TM have also contributed to the various editorial comments and to preparing the manuscript, and histological examination. JS contributed to the surgical segment of the manuscript and was the main attending neurosurgeon for this case. RG contributed the neuro-radiology included with the manuscript. All authors read and approved the final manuscript.
